# Knowledge, attitude, and practice toward hyperuricemia among healthcare workers in Shandong, China

**DOI:** 10.7717/peerj.17926

**Published:** 2024-10-01

**Authors:** Honghai Peng, Ke Zhang, Chunxue Zhang, Jun Gao

**Affiliations:** 1 Department of Neurosurgery, Central Hospital Affiliated to Shandong First Medical University, Jinan, Shandong, China; 2 Department of Anesthesia, Central Hospital Affiliated to Shandong First Medical University, Jinan, Shandong, China; 3 Shandong International Talent Exchange & Service Center, Jinan, Shandong, China

**Keywords:** Knowledge, Attitudes, Practice, Hyperuricemia, Cross-sectional study, Gout

## Abstract

**Background:**

Hyperuricemia is a relatively common condition, with a prevalence of over 20% among the general population. Also, most patients initially present no symptoms. This study aimed to investigate the knowledge, attitude, and practice (KAP) toward hyperuricemia among healthcare workers in Shandong, China.

**Methods:**

Healthcare workers were recruited in this cross-sectional study conducted in Shandong in December 2022. A self-designed questionnaire was used to collect demographic information and KAP data.

**Results:**

A total of 372 questionnaires were distributed, and 216 (58.06%) valid questionnaires were collected from 131 physicians, 80 nurses, and five other healthcare workers. The participants had a mean score of 10.76 ± 2.53 (possible range: 0–14, 76.9%) and 31.94 ± 2.58 (possible range: 0–40, 79.9%) in knowledge and attitude, respectively. The physicians’ and nurses’ practice scores were 47.57 ± 5.34 (possible range: 0–55, 86.5%) and 30.06 ± 4.11 (possible range: 0–35, 85.9%), respectively. The attitude scores were independently associated with proactive practice in both physicians (*P* < 0.001) and nurses (*P* = 0.046).

**Conclusion:**

This study found that healthcare workers in Shandong had adequate knowledge, positive attitudes, and proactive practices towards hyperuricemia. However, there is room for improvement in the attitudes of both physicians and nurses to achieve better practice.

## Introduction

Serum uric acid (SUA) is the end-product of purine metabolism in humans, excreted primarily through the kidneys (>70%) (Ashiq et al., 2021; Schlesinger, 2005). Hyperuricemia is defined as SUA levels of >6 mg/dL (360 μmol/L) and >7 mg/dL (420 μmol/L) in women and men, respectively (Li et al., 2019; von Lueder et al., 2015). The prevalence in the general population is 21% *vs*. 25% in hospitalized individuals. Also, most patients with hyperuricemia are asymptomatic (George & Minter, 2022). According to The Global Burden of Disease (GBD) 2019 database, the global age-standardized prevalence is 652.2 per 100,000 individuals, varying from 183.8 in Central Latin America to 1,722.4 in high-income North America (Han et al., 2024). In China, the prevalence of hyperuricemia from 2000 to 2014 was 13.3% (Liu et al., 2015). People of Asian ancestry are at a three-fold higher risk of hyperuricemia and gout than Caucasians (Butler, Alghubayshi & Roman, 2021). Following the sustained elevation of SUA, one-third of patients with hyperuricemia are affected by gout, characterized by the formation and deposition of monosodium urate crystals around joints (Chen-Xu et al., 2019; Dalbeth et al., 2015). Gout is the most common form of inflammatory arthritis worldwide, affecting 1–6.8% of the population (Dehlin, Jacobsson & Roddy, 2020). This condition imposes a significant economic burden on the patients and the healthcare systems due to treatments, consultations, complications, hospitalizations, and mortality (Degli Esposti et al., 2016; Rai et al., 2015). Gout and hyperuricemia are often treated with urate-lowering therapy (ULT) such as xanthine oxidase inhibitors, uricosuric drugs, uricolytic enzymes, losartan, and sodium-glucose cotransporter-2 inhibitors (Dalbeth, Merriman & Stamp, 2016; FitzGerald et al., 2020; Richette et al., 2017), although the recent guidelines advise against the routine treatment of asymptomatic hyperuricemia in the absence of related symptoms or conditions (FitzGerald et al., 2020). Lifestyle modifications such as weight loss, regular exercise, and dietary changes can also reduce SUA levels and the risk of gout (Choi, 2010). However, whether asymptomatic hyperuricemia should be treated and the appropriate threshold for initiating ULT are still debatable (Skoczynska et al., 2020).

Recently, the role of SUA in various health conditions has gained increasing interest. Despite most asymptomatic patients tend not to develop changes caused by the accumulation of urate crystals, elevated SUA levels have been found to cause silent tissue damage, leading to several diseases such as hypertension, dyslipidemia, obesity, metabolic syndrome, type 2 diabetes, cardiovascular disease, and chronic kidney disease (CKD) (Benn et al., 2018; Chen, Lu & Yao, 2016), but such evidence was not considered enough to recommend drug treatments in asymptomatic hyperuricemia (FitzGerald et al., 2020). On the other hand, impaired renal excretion is responsible for 90% of hyperuricemia, and approximately 50% of CKD patients already have hyperuricemia before undergoing hemodialysis treatment (Hyndman, Liu & Miner, 2016; Johnson et al., 2013; Maiuolo et al., 2016; Ohno, 2011). Furthermore, hyperuricemia has been associated with increased mortality in patients with coronary artery disease, chronic obstructive pulmonary disease, or cancer (Shin et al., 2006; Zhang et al., 2015). In China, hyperuricemia has become the second most common metabolic disease, following diabetes mellitus (Multidisciplinary Expert Task Force on Hyperuricemia and Related Diseases, 2017). The prevalence of hyperuricemia in the Chinese adult population was 11.1% in 2015–16 and 14.0% in 2018–19, indicating an alarming trend (Zhang et al., 2021).

Knowledge, attitude, and practice (KAP) surveys are commonly used in the health sciences to examine what people know, believe, and do about a topic of interest (Andrade et al., 2020). Healthcare workers have a critical role in diagnosing and treating hyperuricemia, and their ability to react systematically to the disease is influenced by their knowledge, attitude, and practice. Since the KAP is influenced by local culture, policies, education, healthcare services available, and guidelines, it is highly variable among countries and even provinces within a given country. Thus, there is a need to assess the understanding of hyperuricemia and its management by healthcare workers in China, as no such study has yet been conducted. Therefore, this study aimed to explore the knowledge, attitude, and practice of hyperuricemia among healthcare workers managing hyperuricemia patients in Shandong, China. We hypothesized that this study might identify knowledge gaps in hyperuricemia diagnosis and treatment, providing insights into improving hyperuricemia management in China.

## Materials and Methods

### Patient and public involvement

Patients and/or the public were not involved in the design, conduct, reporting, or dissemination plans of this research.

### Study design and participants

The cross-sectional study was conducted in seven hospitals in Shandong, China, in December 2022 among healthcare. The inclusion criteria were: (1) Healthcare workers who may treat, care, or communicate with patients with hyperuricemia; (2) with a qualification certificate. Those on leave, in training or education, or refusing to participate were excluded.

This study was approved by the Ethics Committee of Jinan Central Hospital (approval No. 2022-234-01). All participants signed informed consent forms.

### Questionnaire

A self-designed questionnaire was created according to a previous study and guidelines for diagnosing and treating hyperuricemia (Chinese Society of Endocrinology, 2020; Tiwaskar & Sholapuri, 2021). A small-scale pilot test with 30 physicians and 44 other medical workers was conducted, reaching Cronbach’s α coefficients of 0.859 and 0.897, respectively.

The questionnaire contained four dimensions: demographic characteristics (*e.g*., age, sex, residency, ethnicity, education, job title, *etc*.), knowledge, attitude, and practice. The knowledge dimension contained 15 questions and was scored between 0 and 14, with one point for each correct answer and 0 points for each incorrect or unclear answer from K1 to K14. K15 was used for testing questionnaire validity. The attitude category contained eight items evaluated on a five-point Likert scale. Besides items A4 and A6 that were reverse scored, all other items were evaluated from “strongly agree” (five points) to “strongly disagree” (one point). The total scores ranged from 8 to 40. The practice dimension consisted of 11 questions evaluated on a five-point Likert scale, from “always” to “never”, *i.e*., 5–1. P1 to P11 applied to physicians with scores ranging from 11 to 55, while P5 to P11 applied to nurses and other healthcare workers with scores ranging from 7 to 35. The knowledge (>9), attitude (>27), and practice (>38 for physicians and >24 for nurses) scores were considered adequate, positive, and proactive according to the cutoff of 70% of the total score.

Electronic questionnaires were distributed *via* Wechat (Tencent, China) using a QR code created by “Sojump” (Changsha Ranxing Information Technology Co., Ltd) (Guo et al., 2021). A QR code and link to the electronic questionnaire were generated and distributed to the leaders of the seven participating hospitals *via* WeChat, who would then send them to potential participants by WeChat group of their hospital to invite the healthcare workers to participate in the study. The leaders ensured they sent the questionnaire to eligible participants based on the eligibility criteria. Since the potential study population at the seven hospitals was about 4,000, convenience sampling was performed. As the QR code and link were distributed by leaders, those who had access to the questionnaire met the criteria. Informed consent was on the first page of the questionnaire. The participants had to sign the informed consent page to access the questionnaire. The website provided instructions on how to complete the questionnaire. The investigators could be reached through WeChat if needed. The participants could complete the questionnaire at any time during the study period, but only once. If the participant did not complete the questionnaire, a reminder was sent 15 days after the first one. Each IP address could only be used once to submit a questionnaire, and all items had to be completed to ensure the quality and integrity of the questionnaire results.

### Sample size

The sample size was estimated based on the sample size determination method for quantitative surveys, *i.e*., 5–10 participants for each KAP item (Ni, Chen & Liu, 2010). This study had 33 KAP items. Therefore, the minimal sample size was 165–330.

### Statistical analysis

Stata 17.0 was used for the statistical analysis (Stata Corporation, College Station, TX, USA). All continuous variables were normally distributed, expressed as mean ± standard deviation (SD), and compared by one-way analysis of variance or t-test. Categorical data were expressed as frequency (percentage). Multivariable logistic regression analysis was performed to identify the factors associated with the proactive practice of physicians and nurses respectively. A structural equation modeling (SEM) analysis was used to examine how the KAP dimensions influenced each other. A two-sided *P* < 0.05 was considered statistically significant.

## Results

A total of 372 questionnaires were distributed, and 156 questionnaires were excluded because of incomplete responses, inconsistencies in questionnaire responses, and duplicate IP addresses, resulting in 216 valid questionnaires from 131 physicians, 80 nurses, and five other healthcare workers. Table S1 shows the demographic characteristics and KAP scores of the 216 respondents. The knowledge and attitude scores were 10.76 ± 2.53 (76.9%) and 31.94 ± 2.58 (79.9%), respectively. The physicians’ and nurses’ practice scores were 47.57 ± 5.34 (86.5%) and 30.06 ± 4.11 (85.9%), respectively.

Knowledge scores significantly differed by job title (*P* = 0.008), experience in managing hyperuricemia patients (*P* = 0.001), and occupation (*P* < 0.001, Table S1). Participants scored the lowest on the threshold level of SUA (correct rates <70%, Table S2). Only experience managing hyperuricemia patients was associated with a more positive attitude (*P* < 0.001, Table S1). It appears that participants placed too much emphasis on the patient’s responsibility to control SUA levels, as the positive attitude rate was only 40.7% (34.7% + 6%; Table S3). Physicians with a Bachelor’s degree or higher (*P* = 0.008), senior job titles (*P* = 0.007), and experience with hyperuricemia patients (*P* = 0.001) had better practice than their counterparts (Table S1). Most participants encouraged patients to have regular checkups, make lifestyle changes, and participate in educational programs, while 54.7% considered drug therapy or sodium bicarbonate therapy an option (Table S4). Compared with nurses, physicians demonstrated better knowledge (11.33 ± 52.24 *vs*. 9.85 ± 2.69, *P* < 0.001) and comparable attitudes (32.12 ± 2.49 *vs*. 31.60 ± 2.69, *P* = 0.306) (Table S1).

Multivariable logistic regression analysis showed that the attitude scores were independently associated with proactive practice in both physicians (OR (95%CI) = 1.432 [1.173–1.748], *P* < 0.001) and nurses (OR (95%CI) = 1.289 [1.004–1.655), *P* = 0.046] (Tables 1 and 2).

**Table 1:** Multivariate logistic regression analysis on the practice of the physicians (*n* = 131).

Characteristics	Multivariate logistic regression	
	OR (95% CI)	*P*
**Knowledge score**	1.212 [0.941–1.562]	0.137
**Attitude score**	1.432 [1.173–1.748]	<0.001
**Sex**		
Male	Ref.	
Female	1.520 [0.649–3.560]	0.335
**Age (years)**		
<30	Ref.	
30–40	2.151 [0.263–17.572]	0.475
<40	1.930 [0.160–23.355]	0.605
**Residency**		
Non-urban	Ref.	
Urban	0.922 [0.340–2.504]	0.874
**Education**		
Bachelor’s degree	1.356 [0.516–3.565]	0.537
Master’s degree and higher	Ref.	
**Job title**		
None and junior	Ref.	
Intermediate	0.882 [0.146–5.329]	0.891
Senior	2.359 [0.295–18.862]	0.418
**Work experience (years)**		
<10	Ref.	
10–20	0.711 [0.145–3.488]	0.674
>20	1.285 [0.175–9.417]	0.805
**Treated patients with hyperuricemia**		
Yes	0.819 [0.315–2.130]	0.683
No or unclear	Ref.	

**Table 2:** Multivariate logistic regression analysis on the practice of the nurses (*n* = 80).

Characteristics	Multivariate logistic regression	
	OR (95%CI)	*P*
**Knowledge score**	1.212 [0.943–1.556]	0.133
**Attitude score**	1.289 [1.004–1.655]	0.046
**Sex**		
Male	Ref.	
Female	0.499 [0.061–4.067]	0.516
**Age (years)**		
<30	Ref.	
30–40	0.268 [0.033–2.197]	0.475
<40	–	0.999
**Residency**		
Non-urban	Ref.	
Urban	0.814 [0.227–2.915]	0.752
**Job title**		
None and junior	Ref.	
Intermediate	0.611 [0.119–3.145]	0.556
Senior	2.359 [0.076–9.244]	0.886
**Work experience (years)**		
<10	Ref.	
10–20	2.721 [0.420–17.629]	0.294
>20	–	0.999
**Treated patients with hyperuricemia**		
Yes	0.815 [0.245–2.713]	0.739
No or unclear	Ref.	

**Figure 1:**
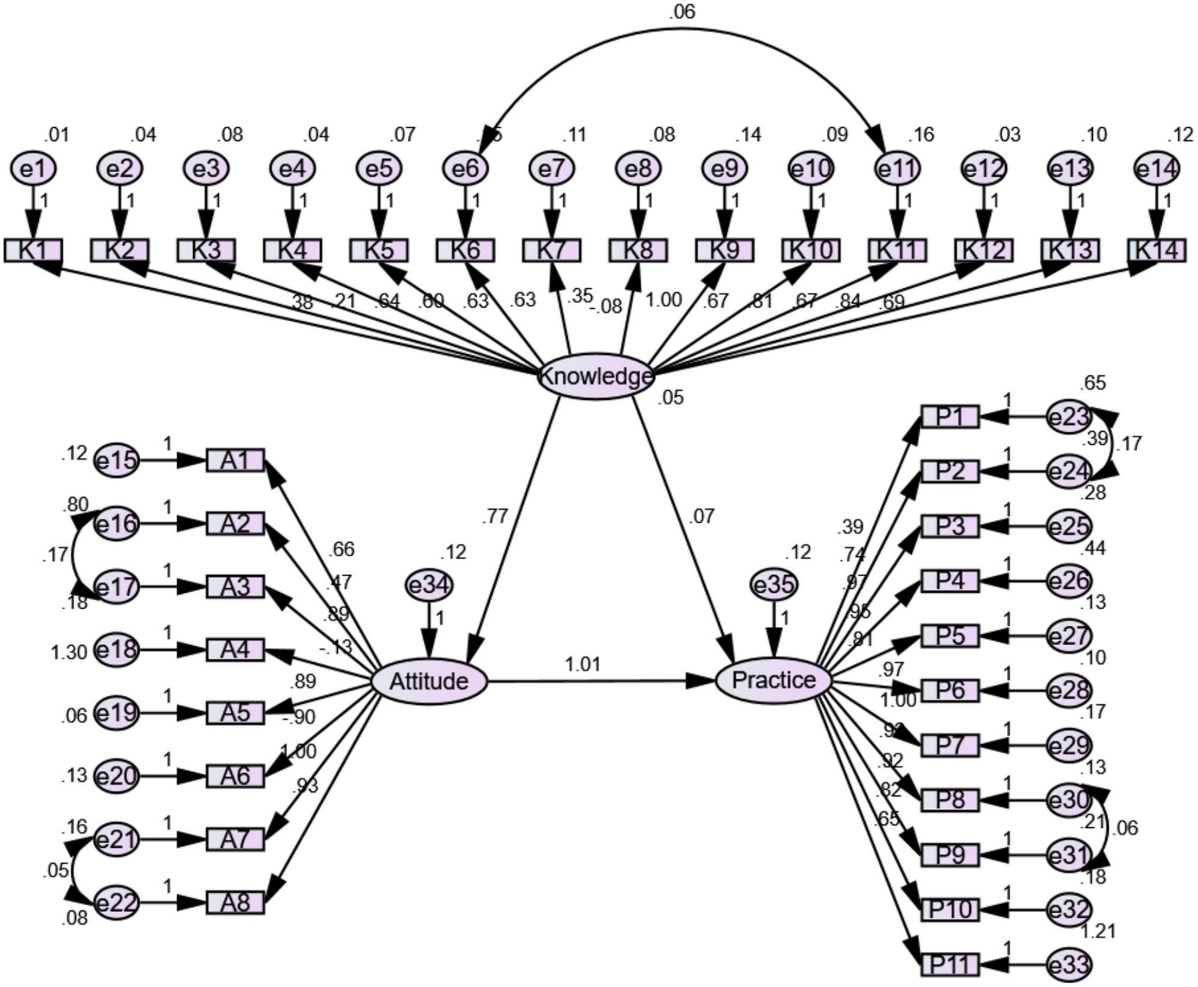
The SEM for physician.

**Figure 2:**
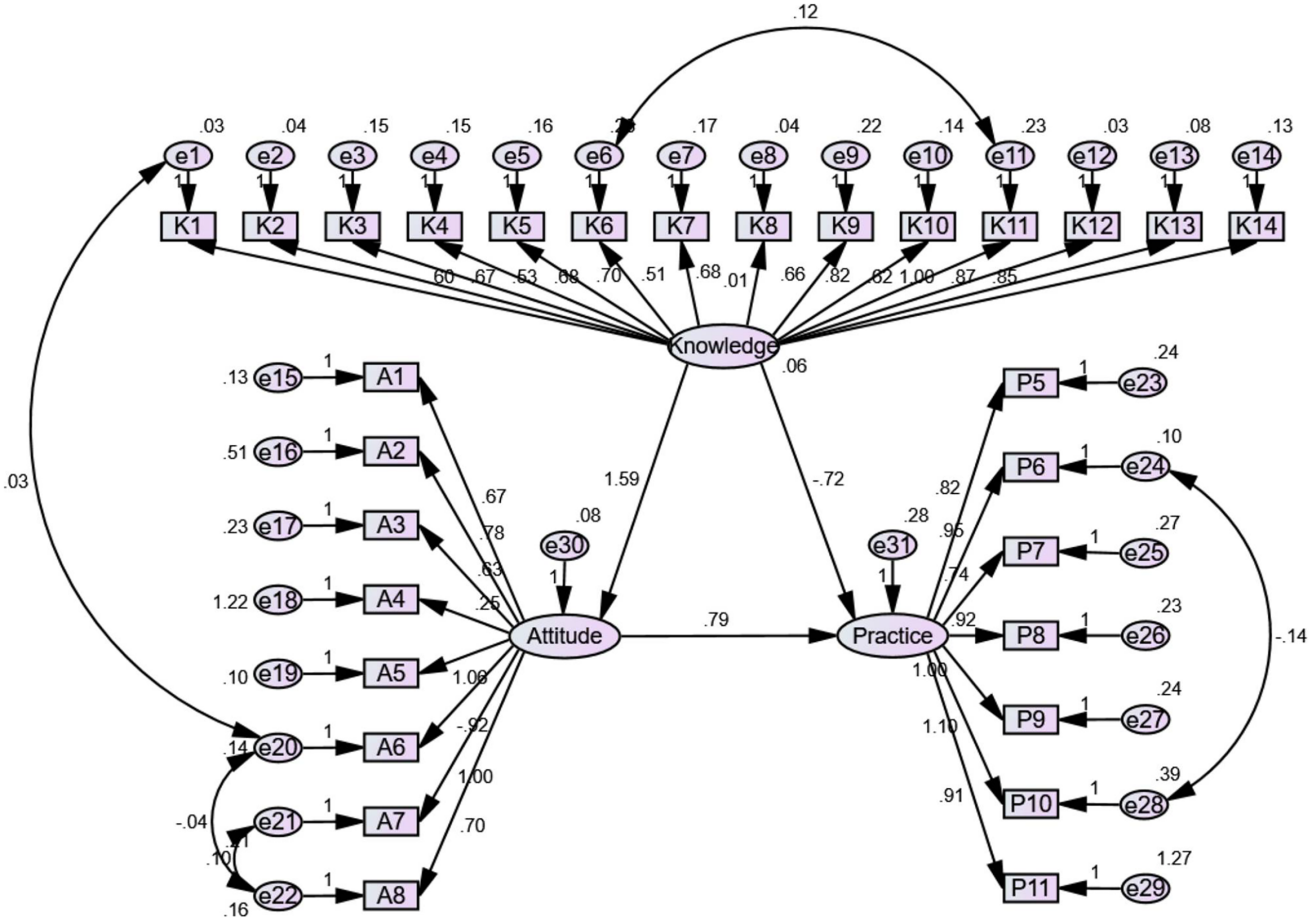
The SEM for nurses.

SEM analyses were performed to examine the interrelationships among the KAP dimensions among physicians (Fig. 1) and nurses (Fig. 2). Among physicians, knowledge positively influenced attitude (β = 0.772, *P* < 0.001) but not practice (β = 0.071, *P* = 0.726), while attitude positively influenced practice (β = 1.011, *P* < 0.001) (Table 3). Among nurses, knowledge positively influenced attitude (β = 1.591, *P* < 0.001) but not practice (β = −0.723, *P* = 0.232), while attitude positively influenced practice (β = 0.793, *P* = 0.013) (Table 3).

**Table 3:** The structural equation modeling (SEM).

			β	*P*
Physicians				
Attitude	<---	Knowledge	0.772	<0.001
Practice	<---	Attitude	1.011	<0.001
Practice	<---	Knowledge	0.071	0.726
Nurses				
Attitude	<---	Knowledge	0.772	<0.001
Practice	<---	Attitude	1.011	<0.001
Practice	<---	Knowledge	0.071	0.726

## Discussion

The present study found that healthcare workers had adequate knowledge, positive attitudes, and proactive practice regarding hyperuricemia. Furthermore, a positive attitude was an independent predictor of good practice among physicians and nurses. These findings provide insight into new ways to improve hyperuricemia management in China.

Physicians who treated hyperuricemia patients before were likely to have better KAP than other participants. Consistent with our findings, greater experience contributed to a better understanding of hyperuricemia management and higher practice scores among primary healthcare providers in the Qassim region of Saudi Arabia (Alraqibah et al., 2022). However, these comparisons must be made with caution, considering the differences in the healthcare systems among countries. Unlike our results, knowledge and practice levels regarding hyperuricemia among primary health providers in Saudi Arabia were very low (45.9% and 23.3%, respectively), which could be due to limited experience resulting from fewer patients or the different dimensions of knowledge involved in the questionnaire (Alqarni & Hassan, 2018; Alraqibah et al., 2022).

Despite positive attitudes towards hyperuricemia management, 97.2% of participants believed that patients must take more responsibility than healthcare workers to control SUA levels. Unfortunately, Huang et al. (2020) identified a lack of education regarding clinical guidelines and disease knowledge and highlighted the importance of cooperation with physicians in diagnosing and treating hyperuricemia in China. Chia (2016) demonstrated that barriers to poorly controlled gout include patient factors such as lack of understanding of the disease and nonadherence to treatment, as well as physical factors such as knowledge gaps and inadequate use of ULT. To bridge the gap, physicians must do more to educate patients, identify treatment targets, and disseminate treatment guidelines (Chia, 2016). Thus, healthcare workers should take a greater role in guiding hyperuricemia diagnosis and treatment than the patients themselves.

Participants scored the least regarding the threshold level of SUA with correct rates <70%. Similarly, only 32.8% of primary health care physicians in Jeddah, Western Region of Saudi Arabia, showed adequate knowledge about asymptomatic hyperuricemia by consideration of the used cutoffs (Alqarni & Hassan, 2018). Medical students from Croatia’s two largest universities also struggled with defining asymptomatic hyperuricemia and treatment goals (Zuzic Furlan et al., 2021), which could be due to the lack of agreement on the definition of hyperuricemia. SUA concentrations >405 µmol/L are a practical value as this represents the urate’s solubility point as measured by automated enzymatic methods in laboratories (Mallat et al., 2016). A few researchers suggest that the values for healthy individuals should be reduced to 300 µmol/L, while others suggest different values for men and women due to the uricosuric effect of estrogenic compounds (Bardin & Richette, 2014; Ferri, 2017; Ramirez & Bargman, 2017). The latest recommendations by the British Society for Rheumatology (BSR) and the European Alliance of Associations for Rheumatology (EULAR) suggest 360 mol/L (6 mg/dL) as the target value when using ULT (Russell et al., 2022). A consensus on hyperuricemia is urgently required to improve healthcare workers’ understanding of this condition.

A retrospective cross-sectional study in Japan showed that ULT was prescribed to 80.7% of gout patients and 72.4% of asymptomatic hyperuricemia patients. However, most patients were given low-dose ULT, and fewer than half of them reached the SUA target (≤420 µmol/L), which may contribute to the 47.8% incidence of gout flares (Koto et al., 2021). According to Chinese guidelines, asymptomatic hyperuricemia should be treated with ULT when SUA reaches 540 or 480 µmol/L in combination with the following comorbidities: hypertension, abnormal lipid metabolism, diabetes, obesity, stroke, coronary heart disease, cardiac insufficiency, urinary acid nephrosis, or damaged renal function (≥CKD stage 2) (Chinese Society of Endocrinology, 2020). A real-world retrospective study at a dedicated gout clinic in China showed that 92.70% of gout patients accepted ULT (Bai et al., 2021); however, the rate of ULT treatment in patients with asymptomatic hyperuricemia remained unclear. In the practice assessment, we found that most healthcare workers encouraged patients’ self-management, such as changing their lifestyles and receiving regular medical examinations. Only about half of the physicians were actively engaged in drug treatment, possibly due to controversy related to the use of ULT in patients with asymptomatic hyperuricemia (Sapankaew et al., 2022).

The SEM analysis showed that knowledge directly influenced attitudes, which in turn directly influenced practice; knowledge had no direct influence on practice, and its influence was, therefore, indirect through attitude. According to the KAP theory, knowledge is the basis underlying practice, but attitude is the force driving practice (Andrade et al., 2020; World Health Organization, 2008). Hence, interventions aiming at improving the knowledge of physicians and nurses toward hyperuricemia should also improve attitude and practice.

Based on our findings, we speculated that hyperuricemia management is suboptimal in Shandong, China. Guidelines and consensus on the diagnosis and management of gout and hyperuricemia should be improved, and stricter compliance with guidelines should be practiced among physicians. Still, to be able to apply guidelines, they must be familiar with the guidelines and their content. Continuing education activities (*e.g*., lectures, online modules, *etc*.) should be designed to improve the knowledge and attitude of the healthcare workers in Shandong, China, which should translate into better practice, *i.e*., better patient care. A better understanding of SUA thresholds and therapies for hyperuricemia is especially needed.

This study has some limitations. It was performed in Shandong, a coastal province in East China. Shandong is a well-established economic center, China’s second most populous province, and the third-largest provincial economy (National Bureau of Statistics of China, 2021). Therefore, the generalizability of the results must be taken with caution, especially in the poorer areas. Indeed, such poorer areas tend to have less accessible healthcare services, which could lead to discrepancies in care received and prognosis (Guo et al., 2020; Zheng et al., 2022). Furthermore, we did not compare the practice between physicians and nurses. In addition, although some pharmacists participated in the study, their KAP was not specifically examined since hospital pharmacists rarely manage patients in the Chinese medical system, especially for hyperuricemia. Since the practice scores of physicians and nurses were independently and positively correlated with attitude scores, and both groups had comparable attitudes toward hyperuricemia, we speculated that their practice levels would also be comparable. The department findings should be interpreted with caution, as the number of participants in each department was small, and some specific professionals could not be identified due to the structure of the medical system. Because this is a cross-sectional study, it is difficult to draw causal conclusions. A SEM was performed as a surrogate of causality, but such results must be taken with caution since causality is statistically inferred rather than observed (Beran & Violato, 2010; Fan, Chen & Shirkey, 2016; Kline, 2023). The questionnaire did not cover some areas, *e.g*., side effects associated with ULT. Considering self-reporting bias, our data may be less reliable than medical records and laboratory measurements. In addition, there is a risk of social desirability bias (Bergen & Labonte, 2020; Latkin et al., 2017). Statistical bias is also possible, but multivariable regression analyses were performed to mitigate confounding. More studies in other areas with larger sample sizes are required to further improve the understanding of the KAP of hyperuricemia.

## Conclusions

Healthcare workers in Shandong, China, demonstrated adequate knowledge, positive attitudes, and proactive practice toward hyperuricemia. A better understanding of SUA thresholds and hyperuricemia drug therapy is needed. Proactive practice can be achieved by improving the attitudes of both physicians and nurses. Future studies could examine the impact of educational activities on the participants’ KAP. The KAP of rheumatologists and pharmacists could also be examined.

## Supplemental Information

10.7717/peerj.17926/supp-1 Supplemental Information 1 Sociodemographic characteristics and KAP scores.

10.7717/peerj.17926/supp-2 Supplemental Information 2 Knowledge questionnaire and correct rates.

10.7717/peerj.17926/supp-3 Supplemental Information 3 Attitude questionnaire and response distribution.

10.7717/peerj.17926/supp-4 Supplemental Information 4 Practice questionnaire and response distribution.

10.7717/peerj.17926/supp-5 Supplemental Information 5 STROBE checklist.

10.7717/peerj.17926/supp-6 Supplemental Information 6 Questionnaire: Chinese.

10.7717/peerj.17926/supp-7 Supplemental Information 7 Questionnaire: English.

10.7717/peerj.17926/supp-8 Supplemental Information 8 Raw data.

## Additional Information and Declarations

### Data Availability

The following information was supplied regarding data availability: Raw data are available in the Supplemental Files.
