# Supplementary material for: Knowledge, attitude, and practice toward hyperuricemia among healthcare workers in Shandong, China
Source: PeerJ. 2024 Oct 1;12:e17926. doi: 10.7717/peerj.17926 (PMC11451443; doi:10.7717/peerj.17926)
Supplement: Supplemental Information 1 [file peerj-12-17926-s001.docx]

**[Supplementary](javascript:;) Table 1**. Sociodemographic characteristics and KAP scores.

| **Variables** | **N (%)** | **Knowledge** | | **Attitude** | | **Practice of physicians (N=131)** | | **Practice of nurses (N=80)** | |
| --- | --- | --- | --- | --- | --- | --- | --- | --- | --- |
|  |  | **Mean ± SD** | **P** | **Mean ± SD** | **P** | **Mean ± SD** | **P** | **Mean ± SD** | **P** |
| **Total** | 216 | 10.76 ± 2.53 |  | 31.94 ± 2.58 |  | 47.57 ± 5.34 |  | 30.06 ± 4.11 |  |
| **Age (years)** |  |  | 0.266 |  | 0.236 |  | 0.067 |  | 0.057 |
| < 30 | 29 (13.4) | 10.21 ± 3.03 |  | 31.62 ± 2.81 |  | 44.85 ± 6.48 |  | 32.25 ± 3.15 |  |
| 30-40 | 86 (39.8) | 10.64 ± 2.64 |  | 31.67 ± 2.74 |  | 47.09 ± 5.04 |  | 29.54 ± 4.06 |  |
| > 40 | 101 (46.8) | 11.02 ± 2.24 |  | 32.26 ± 2.34 |  | 48.38 ± 5.19 |  | 29.48 ± 4.36 |  |
| **Sex** |  |  | 0.097 |  | 0.871 |  | 0.167 |  | 0.323 |
| Mae | 83 (38.4) | 11.12 ± 2.30 |  | 31.98 ± 2.68 |  | 47.01 ± 5.93 |  | 31.67 ± 3.45 |  |
| Female | 133 (61.6) | 10.53 ± 2.64 |  | 31.92 ± 2.52 |  | 48.32 ± 4.38 |  | 29.93 ± 4.15 |  |
| **Residency** |  |  | 0.672 |  | 0.378 |  | 0.816 |  | 0.476 |
| Urban | 153 (70.8) | 10.71 ± 2.53 |  | 32.04 ± 2.50 |  | 47.51 ± 5.33 |  | 29.83 ± 4.44 |  |
| Non-urban | 63 (29.2) | 10.87 ± 2.53 |  | 31.70 ± 2.76 |  | 47.75 ± 5.44 |  | 30.54 ± 3.34 |  |
| **Ethnicity** |  |  | 0.678 |  | 0.560 |  | 0.297 |  |  |
| Han | 214 (99.1) | 10.75 ± 2.52 |  | 31.93 ± 2.59 |  | 47.51 ± 5.35 |  | 30.06 ± 4.11 |  |
| Minorities | 2 (0.9) | 11.50 ± 3.54 |  | 33.00 ± 0.00 |  | 51.50 ± 3.54 |  | - |  |
| **Education** |  |  | 0.060 |  | 0.440 |  | 0.008 |  | 0.179 |
| Associated Degree and lower | 12 (5.6) | 9.42 ± 3.94 |  | 31.50 ± 2.75 |  | 36.00 ± 4.24 |  | 30.90 ± 4.20 |  |
| Bachelor’s Degree | 156 (72.2) | 10.70 ± 2.51 |  | 31.85 ± 2.64 |  | 47.82 ± 5.06 |  | 30.09 ± 4.02 |  |
| Master’s Degree and higher | 48 (22.2) | 11.29 ± 2.02 |  | 32.33 ± 2.31 |  | 47.62 ± 5.42 |  | 25.00 ± 5.66 |  |
| **Job title** |  |  | 0.008 |  | 0.649 |  | 0.007 |  | 0.035 |
| Junior | 49 (22.7) | 9.88 ± 2.97 |  | 31.69 ± 2.65 |  | 47.20 ± 4.80 |  | 31.14 ± 3.82 |  |
| Intermediate | 86 (39.8) | 11.00 ± 2.28 |  | 31.87 ± 2.38 |  | 47.02 ± 4.67 |  | 28.65 ± 4.32 |  |
| Senior | 72 (33.3) | 11.24 ± 1.92 |  | 32.24 ± 2.62 |  | 48.68 ± 5.634 |  | 31.30 ± 3.06 |  |
| None | 9 (4.2) | 9.44 ± 4.61 |  | 31.56 ± 3.75 |  | 39.75 ± 4.99 |  | 32.25 ± 2.99 |  |
| **Department** |  |  | 0.488 |  | 0.582 |  | 0.349 |  | 0.464 |
| Neurology | 6 (2.8) | 11.83 ± 1.60 |  | 33.00 ± 2.45 |  | 47.00 ± 5.70 |  | 32.00 |  |
| Neurosurgery | 20 (9.3) | 10.90 ± 2.53 |  | 31.85 ± 2.72 |  | 46.09 ± 7.84 |  | 30.00 ± 4.44 |  |
| Cardiology | 25 (11.6) | 10.96 ± 1.99 |  | 32.52 ± 2.40 |  | 49.40 ± 3.60 |  | 29.40 ± 4.30 |  |
| Cardiac Surgery | 7 (3.2) | 9.43 ± 4.08 |  | 32.43 ± 2.30 |  | 43.33 ± 5.69 |  | 32.25 ± 2.63 |  |
| Pain Medicine | 19 (8.8) | 11.37 ± 2.03 |  | 31.84 ± 2.46 |  | 49.00 ± 5.15 |  | 34.50 ± 0.71 |  |
| Orthopedic Surgery | 11 (5.1) | 10.45 ± 2.77 |  | 32.36 ± 3.41 |  | 48.30 ± 6.04 |  | 29.00 |  |
| Urology | 15 (6.9) | 10.27 ± 2.25 |  | 30.93 ± 2.31 |  | 46.29 ± 1.98 |  | 28.75 ± 4.74 |  |
| Nephrology | 14 (6.5) | 9.79 ± 3.60 |  | 32.50 ± 2.31 |  | 55.00 |  | 30.54 ± 4.18 |  |
| Endocrinology | 12 (5.6) | 11.17 ± 1.40 |  | 32.75 ± 2.30 |  | 47.57 ± 3.36 |  | 26.20 ± 3.90 |  |
| Gastroenterology | 20 (9.3) | 11.50 ± 1.50 |  | 31.40 ± 2.16 |  | 49.36 ± 3.83 |  | 30.89 ± 2.76 |  |
| Others | 67 (31.0) | 10.58 ± 2.82 |  | 31.69 ± 2.79 |  | 46.55 ± 5.73 |  | 30.33 ± 4.42 |  |
| **Work experience (years)** |  |  | 0.606 |  | 0.198 |  | 0.151 |  | 0.103 |
| < 10 | 55 (25.5) | 10.55 ± 2.67 |  | 32.05 ± 2.65 |  | 46.81 ± 5.44 |  | 31.41 ± 3.28 |  |
| 10 - 20 | 83 (38.4) | 10.70 ± 2.79 |  | 31.55 ± 2.68 |  | 46.85 ± 5.14 |  | 29.17 ± 4.50 |  |
| > 20 | 78 (36.1) | 10.97 ± 2.11 |  | 32.27 ± 2.38 |  | 48.69 ± 5.40 |  | 29.63 ± 4.21 |  |
| **Hospitals classification** |  |  | 0.335 |  | 0.111 |  | 0.103 |  | 0.420 |
| Public primary hospital | 11 (5.1) | 9.73 ± 4.08 |  | 32.73 ± 2.15 |  | 49.14 ± 3.39 |  | 31.33 ± 1.53 |  |
| Public secondary hospital | 40 (18.5) | 11.23 ± 1.70 |  | 32.30 ± 2.21 |  | 48.41 ± 4.48 |  | 29.27 ± 3.58 |  |
| Public tertiary hospital | 144 (66.7) | 10.70 ± 2.64 |  | 31.81 ± 2.66 |  | 47.64 ± 5.79 |  | - |  |
| Specialized hospital | 1 (0.5) | 8.00 |  | 26.00 |  | 39.00 | . | 29.84 ± 4.38 |  |
| Private hospital | 20 (9.3.) | 10.95 ± 1.85 |  | 32.00 ± 2.62 |  | 44.30 ± 3.06 |  | 32.00 ± 3.08 |  |
| **Treated patients with hyperuricemia** |  |  | 0.000 |  | 0.000 |  | 0.001 |  | 0.986 |
| Yes | 114 (52.8) | 11.70 ± 1.67 |  | 32.60 ± 2.30 |  | 48.67 ± 4.14 |  | 30.07 ± 4.04 |  |
| No or unclear | 102 (47.2) | 9.71 ± 2.89 |  | 31.21 ± 2.68 |  | 45.47 ± 6.66 |  | 30.06 ± 4.18 |  |
| **Occupation** |  |  | 0.000 |  | 0.306 |  | - |  |  |
| Physician | 131 (60.6) | 11.33 ± 52.24 |  | 32.12 ± 2.49 |  | - |  | - |  |
| Nurse | 80 (37.0) | 9.85 ± 2.69 |  | 31.60 ± 2.69 |  | - |  | - |  |
| Others | 5 (2.3) | 10.40 ± 3.13 |  | 32.60 ± 2.79 |  | - |  | - |  |
