# Supplementary material for: Knowledge, attitude, and practice toward hyperuricemia among healthcare workers in Shandong, China
Source: PeerJ. 2024 Oct 1;12:e17926. doi: 10.7717/peerj.17926 (PMC11451443; doi:10.7717/peerj.17926)
Supplement: Supplemental Information 2 [file peerj-12-17926-s002.docx]

[**Supplementary**](javascript:;) **Table 2.** **Knowledge questionnaire and correct rates**

| Knowledge, n (%) | Correct rates |
| --- | --- |
| Hyperuricemia is a chronic metabolic disorder associated with abnormal purine metabolism. | 209 (96.8) |
| An individual with hyperuricemia due to congenital metabolic abnormalities may also have obesity, type 2 diabetes, hypertension, and hyperlipidemia, which is termed metabolic syndrome. | 204 (94.4) |
| Acquired diseases (e.g. leukaemia, chronic kidney disease) or drugs that inhibit uric acid excretion (e.g. aspirin) may lead to hyperuricaemia | 183(84.7) |
| In most cases of hyperuricemia, patients have no obvious symptoms other than increased blood uric acid levels, which is referred to as "asymptomatic hyperuricemia". | 188 (87.0) |
| Asymptomatic hyperuricemia may progress to gout. | 182 (84.3) |
| Hyperuricemia is diagnosed by two fasting blood uric acid levels greater than 420 µmol/L on different days. | 149 (69.0) |
| All patients with hyperuricemia should control their weight and exercise regularly while limiting their intake of high-purine and high-fructose foods. | 176 (81.5) |
| It is recommended that patients with hyperuricemia consume more dairy products and fresh vegetables, drink moderate amounts of water, and limit their intake of soy products. | 201 (93.1) |
| In patients with asymptomatic hyperuricemia with uric acid levels ≥ 540 µmol/L or 480 µmol/L and comorbidities, drug therapy is recommended. | 146 (67.6) |
| In addition to its ability to lower uric acid levels, allopurinol is associated with hypersensitivity reactions when used in the Chinese population, and caution should be exercised when administered to patients with chronic kidney disease. | 179 (82.9) |
| Patients suffering from asymptomatic hyperuricemia with or without complications should have their uric acid level maintained within 360/420 µmol/L. | 137 (63.4) |
| Uric acid levels should be monitored regularly in patients with hyperuricemia. | 201 (93.1) |
| A failure to treat hyperuricemia on time can lead to cardiovascular and cerebrovascular damage. | 182 (84.3) |
| Hyperuricemia may damage multiple organs and is causally related to diseases such as kidney stones and chronic kidney disease. | 173 (80.1) |
