# Supplementary material for: Knowledge, attitude, and practice toward hyperuricemia among healthcare workers in Shandong, China
Source: PeerJ. 2024 Oct 1;12:e17926. doi: 10.7717/peerj.17926 (PMC11451443; doi:10.7717/peerj.17926)
Supplement: Supplemental Information 3 [file peerj-12-17926-s003.docx]

**[Supplementary](javascript:;) Table 3. Attitude questionnaire and response distribution**

| Attitude, n (%) | Strongly agree | Agree | Neutral | Disagree | Strongly disagree |
| --- | --- | --- | --- | --- | --- |
| It is important to pay attention to hyperuricemia. | 164 (75.9) | 50 (23.1) | 2 (0.9) | 0 | 0 |
| In patients with asymptomatic hyperuricemia, uric acid levels must be controlled through intervention. | 96 (44.4) | 75 (34.7) | 36 (16.7) | 8 (3.7) | 1 (0.5) |
| There is a greater need for intervention to control uric acid in patients with underlying diseases. | 138 (63.9) | 72 (33.3) | 5 (2.3) | 1 (0.5) | 0 |
| Medications are not necessary to control uric acid in patients with asymptomatic hyperuricemia. | 28 (13.0) | 39 (18.1) | 61 (28.2) | 75 (34.7) | 13 (6.0) |
| A healthy lifestyle is as important as medication to control uric acid | 167 (77.3) | 44 (20.4) | 4 (1.9) | 1 (0.5) | 0 |
| To control uric acid levels, it is more important for patients to take responsibility than healthcare professionals. | 157 (72.7) | 53 (24.5) | 5 (2.3) | 1 (0.5) | 0 |
| Having physicians and nurses participate in the treatment of hyperuricemia at the same time is beneficial to the control of uric acid levels. | 145 (67.1) | 63 (29.2) | 5 (2.3) | 3 (1.4) | 0 |
| Educating patients about hyperuricemia's dangers is the responsibility of medical personnel. | 153 (70.8) | 60 (27.8) | 3 (1.4) | 0 | 0 |
