# Supplementary material for: Knowledge, attitude, and practice toward hyperuricemia among healthcare workers in Shandong, China
Source: PeerJ. 2024 Oct 1;12:e17926. doi: 10.7717/peerj.17926 (PMC11451443; doi:10.7717/peerj.17926)
Supplement: Supplemental Information 4 [file peerj-12-17926-s004.docx]

[**Supplementary**](javascript:;) **Table 4. Practice questionnaire and response distribution**

| Practice, n (%) | Always | Usually | Neutral | Occasionally | Never |
| --- | --- | --- | --- | --- | --- |
| I recommend drug therapy for patients with hyperuricemia.* | 19 (14.5) | 56 (42.7) | 47 (35.9) | 8 (6.1) | 1 (0.8) |
| I modify the treatment strategy if underlying diseases are present.* | 47 (35.9) | 62 (47.3) | 20 (15.3) | 2 (1.5) | 0 |
| I recommend blood uric acid testing for patients with metabolic diseases.* | 75 (57.3) | 42 (32.1) | 13 (9.9) | 0 | 1 (0.8) |
| I recommend that patients with hyperuricemia have their cardiovascular and cerebrovascular systems examined.* | 57 (43.5) | 49 (37.4) | 22 (16.8) | 2 (1.5) | 1 (0.8) |
| I advise patients with hyperuricemia to improve their lifestyles. | 144 (66.7) | 57 (26.4) | 14 (6.5) | 1 (0.5) | 0 |
| I advise patients with hyperuricemia receiving regular checkups at the hospital. | 134 (62.0) | 67 (31.0) | 15 (6.9) | 0 | 0 |
| I am willing to participate in education programs for hyperuricemia patients. | 131 (60.6) | 67 (31.0) | 16 (7.4) | 2 (0.9) | 0 |
| I am responsible for informing patients about the dangers of hyperuricemia in a clear and concise manner. | 128 (59.3) | 74 (34.3) | 11 (5.1) | 3 (1.4) | 0 |
| I pay particular attention to the conditions of the joints of patients with hyperuricemia. | 120 (55.6) | 72 (33.3) | 22 (10.2) | 2 (0.9) | 0 |
| I advise patients with hyperuricemia to consume more water and to urinate more frequently. | 137 (63.4) | 56 (25.9) | 20 (9.3) | 1 (0.5) | 2 (0.9) |
| I advise patients with hyperuricemia to take sodium bicarbonate to alkalize the urine. | 58 (26.9) | 60 (27.8) | 63 (29.2) | 18 (8.3) | 17 (7.9) |

* Only replied by physicians (n=131);
