# Supplementary material for: Knowledge, attitude, and practice toward hyperuricemia among healthcare workers in Shandong, China
Source: PeerJ. 2024 Oct 1;12:e17926. doi: 10.7717/peerj.17926 (PMC11451443; doi:10.7717/peerj.17926)
Supplement: Supplemental Information 6 [file peerj-12-17926-s006.docx]

| **Part I-基本信息** | |
| --- | --- |
| **1.您的年龄：** | 岁 |
| **2.您的性别** | a.男  b.女 |
| **3.您的居住地：** | a.农村  b.城市  c.城郊 |
| **4.您的民族：** | a.汉族  b.少数民族  如您属于少数民族，则您的民族是： |
| **5.您的教育程度：** | a.大专及以下  b.本科  c.硕士及以上 |
| **6.您的职业类型：** | a.医生  b.护士  c.其它： |
| **7.您的职称** | a.初级  b.中级  c.副高  d.正高  e.无职称 |
| **8.您所工作的科室是** | a.神经内科  b.神经外科  c.心内科  d.心外科  e.疼痛科  f.骨外科  g.泌尿外科  h.肾内科  i.内分泌科  j.消化科  k.其它： |
| **9.您的工作年限为：** | 年 |
| **10.您所在的医院类型为** | a.公立一级  b.公立二级  c.公立三级  d.专科医院  e.私立医疗机构 |
| **11.您是否治疗/护理过高尿酸血症患者：** | a.是  b.否  c.不清楚 |

**Part II-知识**

**请您根据您对问题的了解情况选择“对”或“错”。若您不确定问题的答案，请选择“不确定”。**

| **1.****高尿酸血症是一种由嘌呤代谢障碍所引起的慢性代谢疾病** | **a.对** | **b.错** | **c.不确定** |
| --- | --- | --- | --- |
| **2.先天性代谢异常所引起的高尿酸血症常伴有肥胖，二型糖尿病，高血压，高血脂等，临床上称为代谢综合征** |  |  |  |
| **3.后天疾病（如白血病、慢性肾病）或抑制尿酸排泄的药物（如阿司匹林）可能导致高尿酸血症** |  |  |  |
| **4.多数高尿酸血症患者除血液尿酸增高外，通常无明显症状，称为“无症状性高尿酸血症”** |  |  |  |
| **5.无症状性高尿酸血症可能会发展为痛风** |  |  |  |
| **6.高尿酸血症的诊断标准为非同日，2次空腹血尿酸≥420 μmol/L** |  |  |  |
| **7.对所有高尿酸血症患者，都推荐控制体重并保持规律运动，同时限制高嘌呤，高果糖食品的摄入** |  |  |  |
| **8.对高尿酸血症患者，鼓励多摄入多摄入奶制品及新鲜蔬菜，适量饮水，同时限制豆制品的摄入** |  |  |  |
| **9.对尿酸≥540 μmol/L或≥480 μmol/L且有合并症的无症状性高尿酸血症患者，推荐进行药物治疗** |  |  |  |
| **10.别嘌醇具有良好的降尿酸效果，但在中国人群中使用时应关注超敏反应，同时对慢性肾病患者需慎用** |  |  |  |
| **11.对有/无合并症的无症状性高尿酸血症患者，推荐将尿酸控制在360/420 μmol/L以内** |  |  |  |
| **12.高尿酸血症患者需定期检测尿酸水平** |  |  |  |
| **13.高尿酸血症如不及时治疗，可能会危害心脑血管系统** |  |  |  |
| **14.高尿酸血症可能导致多个器官受损，与肾结石、慢性肾病等疾病有明确的因果关系** |  |  |  |
| **15.对所有无症状性高尿酸血症患者，推荐进行药物治疗** |  |  |  |

**Part-III 态度**

**请您根据是否赞同问题中的描述选择“非常同意”到“非常不同意”中的一项。**

| **1.高尿酸血症是一种需要重视的疾病** | **a.非常同意** | **b.同意** | **c.中立** | **d.不同意** | **e.非常不同意** |
| --- | --- | --- | --- | --- | --- |
| **2.无症状性高尿酸血症患者需要通过干预来控制尿酸** |  |  |  |  |  |
| **3.对于合并基础疾病的患者比其它患者更需要通过干预来控制尿酸** |  |  |  |  |  |
| **4.对于无症状性高尿酸血症患者，没必要使用药物来控制尿酸** |  |  |  |  |  |
| **5.对于控制尿酸，养成健康的生活习惯与药物治疗同样重要** |  |  |  |  |  |
| **6.对于控制尿酸，患者自身的行动比医护人员更重要** |  |  |  |  |  |
| **7.医生与护士同时参与到高尿酸血症患者的管理有助于控制尿酸水平** |  |  |  |  |  |
| **8.医护人员有责任让患者意识到高尿酸血症的危害** |  |  |  |  |  |

**Part IV- 实践**

**若您的身份为“医生”，请回答实践部分的第1~11题；若您的身份为“护士”，请回答实践部分的第5~11题。**

| **1.我会建议高尿酸血症患者接受药物治疗** | **a.总是** | **b.经常** | **c.一般** | **d.偶尔** | **e.从不** |
| --- | --- | --- | --- | --- | --- |
| **2.当患者合并基础疾病时，我会改变对患者的治疗策略** |  |  |  |  |  |
| **3.我会推荐有代谢疾病的患者进行血尿酸检查** |  |  |  |  |  |
| **4.我会建议高尿酸血症患者接受心脑血管疾病的检查** |  |  |  |  |  |
| **5.我会建议高尿酸血症患者改善生活习惯** |  |  |  |  |  |
| **6.我会建议高尿酸血症患者定期回医院复查** |  |  |  |  |  |
| **7.我愿意参加对高尿酸血症患者的宣教活动** | **a.非常愿意** | **b.愿意** | **c.一般** | **d.不愿意** | **e.非常不愿意** |
| **8.我会明确告知患者高尿酸血症的危害** | **a.总是** | **b.经常** | **c.一般** | **d.偶尔** | **e.从不** |
| **9.我会关注高尿酸血症患者的关节情况** |  |  |  |  |  |
| **10.我会建议高尿酸血症患者增加饮水及排尿** |  |  |  |  |  |
| **11.我会建议高尿酸血症患者服用小苏打以碱化尿液** |  |  |  |  |  |
