# Supplementary material for: Knowledge, attitude, and practice toward hyperuricemia among healthcare workers in Shandong, China
Source: PeerJ. 2024 Oct 1;12:e17926. doi: 10.7717/peerj.17926 (PMC11451443; doi:10.7717/peerj.17926)
Supplement: Supplemental Information 7 [file peerj-12-17926-s007.docx]

| **Part I-Basic information** | |
| --- | --- |
| **1.Your age:** | Years old |
| **2.Your gender:** | a.Male  b.Female |
| **3.Your residence:** | a.Rural  b.Urban  c.Suburb |
| **4.Your ethnicity:** | a.Han Chinese  b.Ethnic Minority  If you belongs to a minority group, your ethnicity is： |
| **5.Your education:** | a.Junior college and below  b.Undergraduate  c.Postgraduate and above |
| **6.Your type of occupation:** | a.Doctor  b.Nurse  c.Other: |
| **7. Your professional title** | a.Junior  b.Intermediate  c. Vice senior  d.Senior  e.No title |
| **8. The department you work in is** | a.Neurology  b. Neurosurgery  c.Cardiology  d. Cardiac Surgery  e. Pain Medicine  f. Orthopaedic Surgery  g. Urology  h. Nephrology  i.Endocrinology  j.Gastroenterology  k.Other: |
| **9. Duration of your work experience:** | Years |
| **10. The type of hospital you are working in is** | a.Public primary  b.Public secondary  c.Public tertiary  d. Specialist hospital  e. Private medical institution |
| **11. Have you ever treated/cared for a patient with hyperuricemia:** | a.Yes  b.No  c.Unclear |

**Part II-Knowledge**

**Please select " Correct" or "Wrong" depending on your comprehension of the question. If you are unsure of the answer to a question, please select“Unsure”.**

| **Hyperuricemia is a chronic metabolic disorder associated with abnormal purine metabolism.** | **a.Correct** | **b.Wrong** | **c.Unsure** |
| --- | --- | --- | --- |
| **An individual with hyperuricemia due to congenital metabolic abnormalities may also have obesity, type 2 diabetes, hypertension, and hyperlipidemia, which is termed metabolic syndrome.** | **a.Correct** | **b.Wrong** | **c.Unsure** |
| **Acquired diseases (e.g. leukaemia, chronic kidney disease) or drugs that inhibit uric acid excretion (e.g. aspirin) may lead to hyperuricaemia** | **a.Correct** | **b.Wrong** | **c.Unsure** |
| **In most cases of hyperuricemia, patients have no obvious symptoms other than increased blood uric acid levels, which is referred to as "asymptomatic hyperuricemia".** | **a.Correct** | **b.Wrong** | **c.Unsure** |
| **Asymptomatic hyperuricemia may progress to gout.** | **a.Correct** | **b.Wrong** | **c.Unsure** |
| **Hyperuricemia is diagnosed by two fasting blood uric acid levels greater than 420 µmol/L on different days.** | **a.Correct** | **b.Wrong** | **c.Unsure** |
| **All patients with hyperuricemia should control their weight and exercise regularly while limiting their intake of high-purine and high-fructose foods.** | **a.Correct** | **b.Wrong** | **c.Unsure** |
| **It is recommended that patients with hyperuricemia consume more dairy products and fresh vegetables, drink moderate amounts of water, and limit their intake of soy products.** | **a.Correct** | **b.Wrong** | **c.Unsure** |
| **In patients with asymptomatic hyperuricemia with uric acid levels ≥ 540 µmol/L or 480 µmol/L and comorbidities,** **drug therapy is recommended.** | **a.Correct** | **b.Wrong** | **c.Unsure** |
| **In addition to its ability to lower uric acid levels, allopurinol is associated with hypersensitivity reactions when used in the Chinese population, and caution should be exercised when administered to patients with chronic kidney disease.** | **a.Correct** | **b.Wrong** | **c.Unsure** |
| **Patients suffering from asymptomatic hyperuricemia with or without complications should have their uric acid level maintained within 360/420 µmol/L.** | **a.Correct** | **b.Wrong** | **c.Unsure** |
| **Uric acid levels should be monitored regularly in patients with hyperuricemia.** | **a.Correct** | **b.Wrong** | **c.Unsure** |
| **A failure to treat hyperuricemia on time can lead to cardiovascular and cerebrovascular damage.** | **a.Correct** | **b.Wrong** | **c.Unsure** |
| **Hyperuricemia may damage multiple organs and is causally related to diseases such as kidney stones and chronic kidney disease.** | **a.Correct** | **b.Wrong** | **c.Unsure** |
| **For all patients with asymptomatic hyperuricemia, drug therapy is recommended.** | **a.Correct** | **b.Wrong** | **c.Unsure** |

**Part III-Attitude**

**Please choose from “Strongly agree” to “Strongly disagree” depending on whether you agree with the description in the question.**

| **It is important to pay attention to hyperuricemia.** | **a.Strongly agree** | **b.Agree** | **c.Neutral** | **d.Disagree** | **e.Strongly disagree** |
| --- | --- | --- | --- | --- | --- |
| **In patients with asymptomatic hyperuricemia, uric acid levels must be controlled through intervention.** | **a.Strongly agree** | **b.Agree** | **c.Neutral** | **d.Disagree** | **e.Strongly disagree** |
| **There is a greater need for intervention to control uric acid in patients with underlying diseases.** | **a.Strongly agree** | **b.Agree** | **c.Neutral** | **d.Disagree** | **e.Strongly disagree** |
| **Medications are not necessary to control uric acid in patients with asymptomatic hyperuricemia.** | **a.Strongly agree** | **b.Agree** | **c.Neutral** | **d.Disagree** | **e.Strongly disagree** |
| **A healthy lifestyle is as important as medication to control uric acid** | **a.Strongly agree** | **b.Agree** | **c.Neutral** | **d.Disagree** | **e.Strongly disagree** |
| **To control uric acid levels, it is more important for patients to take responsibility than healthcare professionals.** | **a.Strongly agree** | **b.Agree** | **c.Neutral** | **d.Disagree** | **e.Strongly disagree** |
| **Having physicians and nurses participate in the treatment of hyperuricemia at the same time is beneficial to the control of uric acid levels.** | **a.Strongly agree** | **b.Agree** | **c.Neutral** | **d.Disagree** | **e.Strongly disagree** |
| **Educating patients about hyperuricemia's dangers is the responsibility of medical personnel.** | **a.Strongly agree** | **b.Agree** | **c.Neutral** | **d.Disagree** | **e.Strongly disagree** |

**Part IV-Practice**

**If you are a 'doctor', please answer Questions 1-11 in this practice section; if you are a 'nurse', please answer Questions 5-11 in this practice section.**

| **I recommend drug therapy for patients with hyperuricemia.*** | **a.Always** | **b.Usually** | **c.Neutral** | **d.Occasionally** | **e.Never** |
| --- | --- | --- | --- | --- | --- |
| **I modify the treatment strategy if underlying diseases are present.*** | **a.Always** | **b.Usually** | **c.Neutral** | **d.Occasionally** | **e.Never** |
| **I recommend blood uric acid testing for patients with metabolic diseases.*** | **a.Always** | **b.Usually** | **c.Neutral** | **d.Occasionally** | **e.Never** |
| **I recommend that patients with hyperuricemia have their cardiovascular and cerebrovascular systems examined.*** | **a.Always** | **b.Usually** | **c.Neutral** | **d.Occasionally** | **e.Never** |
| **I advise patients with hyperuricemia to improve their lifestyles.** | **a.Always** | **b.Usually** | **c.Neutral** | **d.Occasionally** | **e.Never** |
| **I advise patients with hyperuricemia receiving regular checkups at the hospital.** | **a.Always** | **b.Usually** | **c.Neutral** | **d.Occasionally** | **e.Never** |
| **I am willing to participate in education programs for hyperuricemia patients.** | **a.Always** | **b.Usually** | **c.Neutral** | **d.Occasionally** | **e.Never** |
| **I am responsible for informing patients about the dangers of hyperuricemia in a clear and concise manner.** | **a.Always** | **b.Usually** | **c.Neutral** | **d.Occasionally** | **e.Never** |
| **I pay particular attention to the conditions of the joints of patients with hyperuricemia.** | **a.Always** | **b.Usually** | **c.Neutral** | **d.Occasionally** | **e.Never** |
| **I advise patients with hyperuricemia to consume more water and to urinate more frequently.** | **a.Always** | **b.Usually** | **c.Neutral** | **d.Occasionally** | **e.Never** |
| **I advise patients with hyperuricemia to take sodium bicarbonate to alkalize the urine.** | **a.Always** | **b.Usually** | **c.Neutral** | **d.Occasionally** | **e.Never** |

*: The question was for doctors only.
